# Diversity and functional profile of gut symbiotic bacteria between *Lysinibacillus sphaericus* C_3_-41 susceptible and resistant *Culex quinquefasciatus* Say as revealed by 16S rRNA gene high-throughput sequencing

**DOI:** 10.3389/fmicb.2022.991105

**Published:** 2022-11-03

**Authors:** Xiaolei Zhang, Haoran Meng, Xiaomin Hu, Zhiming Yuan

**Affiliations:** ^1^Hubei Engineering Technology Research Center for Forewarning and Management of Agricultural and Forestry Pests, College of Agriculture, Yangtze University, Jingzhou, China; ^2^Key Laboratory of Special Pathogens and Biosafety, Wuhan Institute of Virology, Chinese Academy of Sciences, Wuhan, Hubei, China

**Keywords:** *Culex quinquefasciatus*, *Lysinibacillus sphaericus* C3–41, gut symbiotic bacteria, resistance, 16S rRNA

## Abstract

Previous studies have demonstrated that symbiotic gut bacteria in insects are involved in the development of insecticide resistance, but the relationship between the symbiotic gut bacteria and resistance to *Lysinibacillus sphaericus* C_3_-41 in *Culex pipiens quinquefasciatus* remains unclear. In this study, the abundance and diversity of gut symbionts of *Cx*. *quinquefasciatus* that were resistant (RLCql) or susceptible (SLCql) to *L. sphaericus* C_3_-41 were analyzed by high-throughput 16S rRNA sequencing. The main phyla among the symbiotic gut bacterial communities of *Cx*. *quinquefasciatus* were Proteobacteria, Actinobacteria, Firmicutes, and Bacteroidetes. However, the relative abundance of Firmicutes, Proteobacteria, and unidentified Bacteria in the gut of the resistant strain of *Cx*. *quequinfasciatus* (RLCql colony) was higher compared to the susceptible strain (SLCql colony). The NMDS (Non-Metric Multi-Dimensional Scaling) and unweighted unifrac PCoA analyses also revealed significant differences between the symbiotic gut bacterial communities from the resistant and susceptible strains, suggesting that bacterial insecticides can alter bacterial composition. Ultimately, the changes in the bacterial community likely occurred after the development of resistance to *L. sphaericus* C_3_-41. These results provide guidance for further research into the mechanisms of gut symbionts involved in resistance against *L*. *sphaericus* C_3_-41 in *Cx*. *quinquefasciatus*.

## Introduction

*Culex quinquefasciatus* Say (Diptera: Culicidae) is an important medical insect that is distributed throughout the world (Samy et al., 2016; Zhang et al., 2019a). *Cx. quinquefasciatus* breed better in water that is contaminated with organic debris, such as rotten vegetation or human and animal excrement (Shah et al., 2016). Therefore, the larvae of this species are often discovered in urban sewer systems, irrigation ditches, cesspools, septic tanks, used tires, or containers of non-potable water (Shah et al., 2016; Zhang et al., 2019b; Díaz-Nieto et al., 2021). *Cx. quinquefasciatus* is the primary vector of Japanese encephalitis virus (JEv), St. Louis encephalitis virus (SLEv), West Nile virus (WNv), Western equine encephalitis virus (WEEv), Rift Valley fever virus (RVF), and the filarial nematode (Reid et al., 2018; Zhang et al., 2019a). These pathogens may be transmitted when humans are bitten during the day or night (Guo et al., 2016). These vector-borne diseases pose a major threat to global health and increase the economic burden of individuals and families (Mainali et al., 2017; Redekop et al., 2017). It is widely accepted that these diseases can be controlled or eliminated by targeting vector mosquitoes (Zhang et al., 2019b); thus, insecticides are extensively and intensively used to control *Cx. quinquefasciatus* worldwide (Yang et al., 2021). However, widespread broad-spectrum insecticide usage has given rise to concerns regarding environmental impact and the evolution of insecticide resistance in mosquitoes. Therefore, the development of bacteria as alternative control agents to insecticides has become highly appealing. Examples may include *Bacillus thuringiensis* and *Lysinibacillus sphaericus* Niede (Yu et al., 2017).

*Lysinibacillus sphaericus* is a globally distributed, aerobic, gram-positive, mesophilic, and spore-forming bacterium (Fu et al., 2017a). *L. sphaericus* is widely distributed in nature, including soil and aquatic environments, and its spores may even recirculate and survive in unusual environments, such as the bodies of dead mosquito larvae (Fu et al., 2017b). *L. sphaericus* produces insecticidal binary (Bin) protoxin as crystalline inclusions during bacterial sporulation, which have high larvicidal activity. Therefore, *L. sphaericus* has been extensively used for controlling the genus *Culex* in China, Vietnam, Thailand, India, Cuba, Denmark, Belgium, France, the USA, and Brazil (Xiong et al., 2010). Unfortunately, due its action on a single toxin receptor within the larval midgut, resistance to *L. sphaericus* in *Culex* has developed quickly in a short period of time (Guo et al., 2013). According to the Arthropod Pesticide Resistance Database (APRD), current field surveys report different levels of resistance to *L. sphaericus* in *Cx. quinquefasciatus* and *Cx. pipiens* populations from China, Thailand, India, France, and Brazil (Yuan et al., 2000; Chenniappan and Ayyadurai, 2012). These studies demonstrated that such high levels of resistance to *L. sphaericus* are predominantly due to the absence and alteration of the functional receptors in the midgut leading to the failure of Bin toxin binding (Guo et al., 2013).

*Lysinibacillus sphaericus* C_3_-41 was isolated in 1987 by the Wuhan institute of Virology, Chinese Academy of Sciences and is highly toxic to *Culex* larvae (Xiong et al., 2010). A *L. sphaericus* C_3_-41 liquid formulation (Jianbao^®^) has been successfully applied to control urban mosquito larvae in China for more than three decades and has demonstrated good efficacy (Xiong et al., 2010). However, under laboratory conditions, after 13 generations of exposure to high concentrations of *L. sphaericus* C_3_-41, a susceptible colony developed high-level resistance against this larvicide (resistance ratio, RR > 140,000-fold) (Pei et al., 2002). Moreover, high-level resistance to this species in a field mosquito colony was also recorded, further hindering the application of this biopesticide in mosquito control (Yuan et al., 2000).

Insects possess diverse symbiotic microbiota in their gut lumen that likely play an essential role in several physiological functions (Wang and Qu, 2017). For example, these symbiotic microorganisms may regulate the growth and development of host insects, as well as their reproduction, aggregation, movement, immune responses, and detoxification mechanisms (Wei et al., 2018). Symbiotic bacteria may also confer chemical insecticide resistance in agricultural pests (Kikuchi et al., 2012; Cheng et al., 2017; Hao et al., 2018; Wang et al., 2021). Conversely, some symbiotic bacteria can invade the body cavity or hemocoel through toxin-induced epithelial lesions and promote the death of medically important insects (Wei et al., 2017). Moreover, shifts in susceptibility to insecticides in insects have been linked with changes in the gut bacterial loads and diversity, as described above.

In this study, we hypothesize that there may be a close relationship between the changes in gut symbionts and *L. sphaericus* C_3_-41 resistance in *Cx. quinquefasciatus*. Using amplicon sequencing, we investigated the gut symbiont composition of the *Cx. quinquefasciatus* in resistant and susceptible populations, with the aim of elucidating the possible role of symbiotic gut bacteria on the development of *L. sphaericus* C_3_-41 resistance in mosquitoes.

## Materials and methods

### Mosquitoes

The susceptible *Cx. quinquefasciatus* (SLCql), which was established from a laboratory-reared colony more than 10 years ago, was reared without exposure to any insecticide. The resistant *Cx. quinquefasciatus* (RLCq1) was derived from the susceptible colony and selection was performed by exposure to *L. sphaericus* C_3_-41 under laboratory conditions, with a resistance ratio of more than 140,000-fold. Larvae of the two colonies were reared in enamel pans filled with dechlorinated tap water and fed with a mixture of yeast powder and wheat mill or cat chow. The pupae were removed from the pans every day and placed in screen cages for emergence. The adults were allowed to feed on 10% sucrose solution, and the females were fed with horse serum purchased from Shanghai Yuanye Biological Technology Co., Ltd., (Shanghai, China). All larvae and adults were held at 27 ± 1°C with a photoperiod of 12:12 h (light-dark).

### *Lysinibacillus sphaericus* C_3_-41

The *L. sphaericus* C_3_-41 was grown on an MBS (medium *Bacillus sphaericus*) medium plate at 30°C for 72–120 h or in MBS (medium *B*. *sphaericus*) at 30°C for 36 h. The bacteria were scraped off and rinsed with phosphate buffer before freeze-drying into powder. The MBS medium contained 6.8 g KH_2_PO_4_, 0.3 g MgSO_4_⋅7H_2_O, 0.2 g CaCl_2_⋅2H_2_O, 0.002 g MnSO_4_⋅H_2_O, 0.002 g Fe_2_(SO_4_)_3_, 0.002 g ZnSO_4_⋅6H_2_O, 10 g tryptone, 2.0 g yeast extract, and 20 g agar powder per liter of medium (pH 7.0).

### Bioassays

Bioassays were carried out according to the standard method recommended by the World Health Organization (Zhang et al., 2019a). Twenty third-instar larvae were transferred into 100 mL of distilled water in a 200 mL plastic cup. Three replicates for each dose of insecticide and nine doses in total were performed, and the controls were treated with dechlorinated tap water. All treatments were maintained at a temperature of 27 ± 1°C and 40–50% relative humidity with a 12 h light/12 h dark photoperiod. Mortality was assessed after exposure to insecticide for 48 h. The LC_50_ values with 95% confidence interval, slopes with standard error (SE), and chi-square value (χ^2^) with degrees of freedom (df) were calculated using a regression model based on a probit transformation of mortalities and a logarithmic transformation of concentrations tested, i.e., a log-probit model by Polo Plus software.

### Tissue collection

The third instar larvae of the SLCq1 and RLCq1 strains were used for sample preparation. These larvae were surface-washed in a sterile petri dish containing 75% ethanol for 90 s, and then rinsed four times with sterilized water. The guts and gut contents from 60 insects were collected under a stereomicroscope on a surface-disinfected bench for each independent biological replicate. Then, the guts and gut contents were transferred into a microcentrifuge tube containing 200 μL phosphate-buffered saline (PBS, pH 7.8) and homogenized. The homogenization buffer was frozen at –80°C before DNA extraction.

The samples for 16S rRNA gene Illumina sequencing were dissected from 50 individuals in each generation for 6 generations; thus, each strain had six independent biological replicates (SLCql1, SLCql2, SLCql3, SLCql4, SLCql5, SLCql6, and RLCql1, RLCql2, RLCql3, RLCql4, RLCql5, and RLCql6).

### DNA extraction from susceptible colony and resistant colony

The total genomic DNA of symbiotic bacteria was extracted from the prepared samples using the QIAamp DNA Stool Mini Kit following the manufacturer’s protocol. DNA concentration was detected using a Qubit^®^ 2.0 (Invitrogen). DNA purity was monitored on 1% agarose gels.

### 16S rRNA high-throughput sequencing

16S rRNA genes from distinct regions (16S V4) were amplified used specific primers (e.g., 16S V4: 515F-806R) with barcodes. All PCR reactions were carried out using the Phusion^®^ High-Fidelity PCR Master Mix (New England Biolabs). Equal volumes of 1 × loading buffer (containing SYBR green) were mixed with PCR products and 2% agarose gel electrophoresis was performed for detection. PCR products were mixed in equidensity ratios. Then, mixed PCR products were purified with the GeneJET™ Gel Extraction Kit (Thermo Scientific). Sequencing libraries were generated using the Ion Plus Fragment Library Kit 48 rxns (Thermo Scientific) following the manufacturer’s recommendations. The library quality was assessed on the Qubit^@^ 2.0 Fluorometer (Thermo Scientific). Finally, the library was sequenced on an Ion S5TM XL platform and 400 bp/600 bp single-end reads were generated.

### Data analysis of 16S rDNA sequences

OTU abundance data were normalized using standard sequence numbers corresponding to the sample with the least sequences. Subsequent analyses of alpha diversity and beta diversity were performed based on the normalized data.

#### Alpha diversity analysis

Alpha diversity describes the complexity of species diversity for a sample. Two indices were used: the observed species and Shannon indices, which were both calculated using QIIME (Version 1.7.0) and illustrated using R software (Version 2.15.3).

#### Beta diversity analysis

Beta diversity analysis was performed to evaluate differences across samples in the context of species complexity. Beta diversity analyses on both weighted and unweighted unifrac distances were calculated using QIIME software (Version 1.7.0).

Principal Coordinate Analysis (PCoA) was performed to obtain the principal coordinates and visualize complex, multidimensional data. A previously obtained weighted or unweighted unifrac distance matrix was transformed to a new set of orthogonal axes, with first principal coordinate representing the factor accounting for the greatest variation, the second principal coordinate representing the next most important factor, and so on. PCoA plots were constructed using the WGCNA package, stat package, and ggplot2 package in R software (Version 2.15.3).

Unweighted Pair-group Method with Arithmetic Means (UPGMA) clustering is a hierarchical clustering method for interpreting the distance matrix using average linkage. UPGMA clustering was conducted with QIIME software (Version 1.7.0).

### Statistical analysis

To identify significant differences between the symbiotic bacteria of SLCql and RLCql, a statistical method introduced in Metastats software was used. A *p*-value of ≤ 0.05 was considered significant.

## Results

### Toxicity of *Lysinibacillus sphaericus* C_3_-41 to *Culex quinquefasciatus*

Bioassay results showed that the *L. sphaericus* C_3_-41 freeze-dried powder had a high activity against the susceptible colony (SLCq1), with a 50% lethal concentration (LC_50_) of 0.0098 mg/L. However, little toxicity was observed in the resistant colony (RLCq1), in which the LC_50_ was greater than 1,000 mg/L. The RLCq1 has a resistance ratio of > 100, 000-fold to C_3_-41 (Table 1).

**TABLE 1:** Toxicity of *Lysinibacillus sphaericus* C_3_-41 (*Ls* C_3_-41) to susceptible (SLCql) and resistant (RLCql) *Culex quinquefasciatus*.

Strain	No.^a^	Slope (SE^b^)	LC_50_^d^ (95% CI^c^) mg/L	χ^2e^ (df)^f^	Resistance ratio
SLCql	540	1.47 (0.18)	0.0098 (0.007–0.013)	5.14 (5)	1.0
RLCql	300	-	> 1,000	-	> 100,000

^a^N: the number of insects in bioassay.

^b^SE: standard error.

^c^CI: confidence limit.

^d^LC_50_: half-lethal concentration.

^e^χ^2^: Chi-square value.

^f^df: degrees of freedom.

-: no value for this parameter.

### 16S rRNA sequencing of gut bacteria

Based on high-throughput sequencing of the V4 hypervariable region of the 16S rRNA gene in gut bacteria, a total of 424, 268 and 503, 677 raw reads were obtained from the guts of *Cx. quinquefasciatus* from SLCql and RLCql, respectively (Table 2). After removing the adaptors, low-quality sequences, overlapping PE reads, and chimeras, an average of 64, 998 and 77, 314 clean reads with an average length of more than 420 bp were obtained from SLCql and RLCql, respectively (Table 2). The greatest number of clean reads was observed in RLCql6, and the fewest clean reads was found in SLCql5 (Table 2).

**TABLE 2:** General statistics of the 16S rRNA gene sequencing for all samples.

Sample	Raw reads	Clean reads	Bases (nt)	AvgLen (nt)	Q20	GC%	Effective%
SLCql1	64074	58032	24666390	425	83.61	53.05	90.57
SLCql2	63759	59655	25335392	424	84.39	53.63	93.56
SLCql3	87039	80056	34112373	426	84.07	53.66	91.98
SLCql4	78309	73403	30519852	415	82.97	54.48	93.74
SLCql5	50799	45892	19194215	418	82.77	53.97	90.34
SLCql6	80288	72950	30800498	422	82.96	53.90	90.86
Total	424268	389988	-	-	-	-	-
Mean	70711.33	64998	27438120	421.67	83.46	53.78	91.84
RLCql1	87479	80144	34089687	425	84.97	52.29	91.62
RLCql2	83317	75745	31695677	418	83.19	51.70	90.91
RLCql3	84472	80071	33540979	418	82.35	52.29	94.79
RLCql4	85812	80330	34201426	425	84.64	52.00	93.61
RLCql5	74702	67384	27855108	413	85.29	54.12	90.20
RLCql6	87895	80208	33918008	422	84.68	53.45	91.25
Total	503677	463882	-	-	-	-	-
Mean	83946.17	77313.67	32550148	420.17	84.19	52.64	92.06

Raw reads, sequences after filtering out low-quality bases; Clean reads, sequences that are finally used for subsequent analysis after filtering out chimeras; Bases, the number of bases in the final Clean reads; AvgLen, the average length of Clean reads; Q20, the percentage of bases with a base mass value > 20 (sequencing error rate < 1%) in Clean reads; GC (%), the content of GC bases in Clean reads; Effective (%), the percentage of the number of Clean reads out of the number of Raw reads.

### Alpha diversity analysis

**FIGURE 1:**
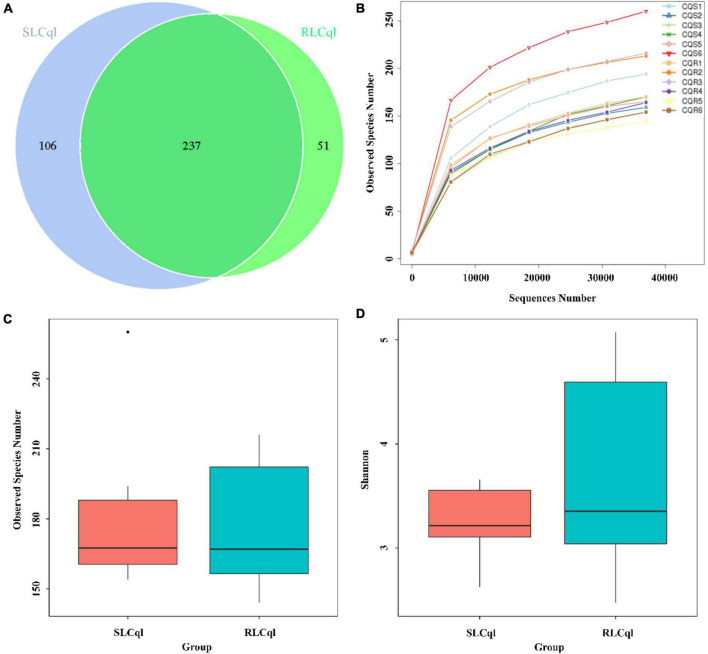
Alpha diversity of symbiotic gut bacteria from strains that are susceptible and resistant to *Lysinibacillus sphaericus* C_3_-41 [**(A)** Venn Graph, **(B)** Rarefaction curve, **(C)** Observed species, **(D)** Shannon index].

Generally speaking, a sequence that clusters into an OTU with more than 97% sequence consistency is considered to be derived from the same species. The most intuitive OTU-based alpha diversity evaluation is the number of OTUs, which can reflect species richness. Analysis of 16S RNA sequencing results showed that 288 and 343 OTUs were attained from the symbiotic gut bacteria of the strains of *Cx. Quinquefasciatus* that were resistant (RLCql) and susceptible (SLCql) to *L. sphaericus* C_3_-41, respectively (Figure 1A). These OTUs corresponded to 176 bacterial species in RLCql and 183 in SLCql (Figures 1B,C). Although a greater number of bacterial species were observed in SLCql than RLCql, this difference was not significant and the abundance distribution of SLCql was also not significantly different to that of RLCql (Figures 1C,D and Table 3). In addition to the number of OTUs, observed species and Shannon indices were used to estimate the total species richness, both of which yielded similar results (Table 3). In addition, the rarefaction curve of the sequencing data tended to be smooth, demonstrating that the sequencing data volume increased at the expected rates. Moreover, the sequencing depth showed good coverage of all species in the sample. Therefore, the sequencing results are a good reflection of the bacteria in the sample, and any further addition of sequencing depth would be unlikely to significantly influence the alpha diversity (Figure 1B). This contrasts many 16S rRNA gene sequencing studies, which ultimately do not obtain smooth rarefaction curves for alpha diversity due to sequencing errors and other reasons.

**TABLE 3:** Alpha diversity indices of gut bacteria from third-instar *Culex quinquefasciatus* strains that are susceptible and resistant to *Lysinibacillus sphaericus* C_3_-41.

Group	Observed species	Shannon
RLCql	176.83 ± 12.45 a	3.70 ± 0.44 a
SLCql	183.67 ± 16.29 a	3.24 ± 0.16 a

The same letters in any one column indicate no significant difference.

### Symbiotic gut bacterial communities

**FIGURE 2:**
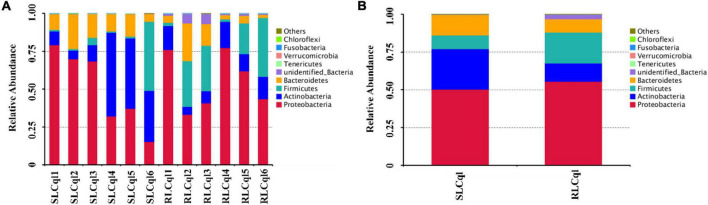
Symbiotic gut bacterial composition at the phylum level in strains that are susceptible and resistant to *Lysinibacillus sphaericus* C_3_-41. **(A)** The taxonomic composition of the dominant phyla per replicate of susceptible and resistant strains of *Cx. quinquefasciatus*, **(B)** the average taxonomic composition of the dominant phylum of the susceptible and resistant strains of *Cx. quinquefasciatus*.

The top 10 phyla with the highest abundance of symbiotic gut bacteria were illustrated in a cumulative histogram of the different bacterial compositions in RLCql and SLCql. The symbiotic gut bacteria of the susceptible colony were mainly comprised of Proteobacteria, Actinobacteria, Firmicutes, and Bacteroidetes (Figures 2A,B), while the resistant colony mainly consisted of Proteobacteria, Actinobacteria, Firmicutes, Bacteroidetes, and unidentified bacteria (Figures 2A,B). The relative abundances of the symbiotic bacterial communities in RLCql were markedly different to those in SLCql, indicating that the gut bacterial flora may have a functional role in the development of insecticide resistance in mosquitoes.

### Beta diversity analysis

**FIGURE 3:**
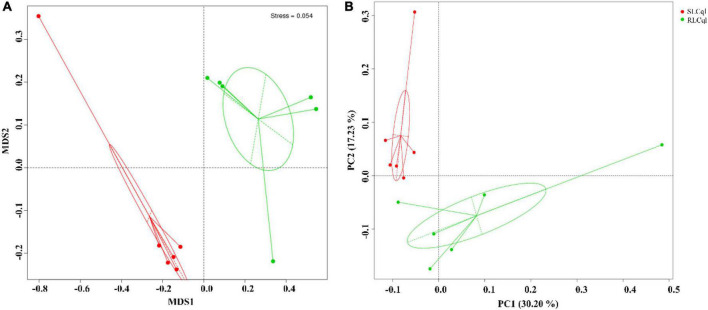
Beta diversity analysis of symbiotic gut bacteria from strains that are susceptible and resistant to *Lysinibacillus sphaericus* C_3_-41. **(A)** Non-metric multidimensional scaling (NMDS), **(B)** unconstrained principle coordinate analysis (PCoA).

The NMDS (Non-Metric Multi-Dimensional Scaling) and unweighted unifrac PCoA (Principal Coordinate Analysis) were used for beta diversity (β-diversity) analysis of the symbiotic gut bacterial communities of RLCql and SLCql (Figure 3A). The results of the NMDS analyses revealed that the symbiotic gut bacterial communities in the resistant strain differed significantly from those of the susceptible strain (Figure 3A). A similar pattern was observed in the PCoA analysis of unweighted unifrac distances (Figure 3B), which showed that the first and second principal coordinates explained 30.20 and 17.23% of the variation in the microbial β-diversity data, respectively. Therefore, the gut bacterial composition was likely altered in the resistant *Cx. quinquefasciatus* (Figure 3B).

**FIGURE 4:**
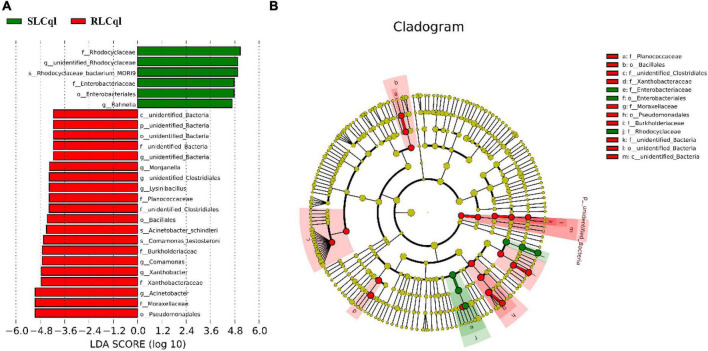
Taxons identified from Line Discriminant analysis (LDA) Effect Size (LEFSe) for strains of *Culex quinquefasciatus* that are susceptible (SLCql) and resistant (RLCql) to *Lysinibacillus sphaericus* C_3_-41. **(A)** Taxonomic cladogram. Taxa enriched in RLCql or SLCql are colored red or green, respectively. **(B)** SLCq1-enriched taxa are shown with a positive LDA score (green), and taxa enriched in RLCql are shown with a negative score (red). Only taxa meeting an LDA significance threshold > 4 and *p* < 0.05 are shown.

**FIGURE 5:**
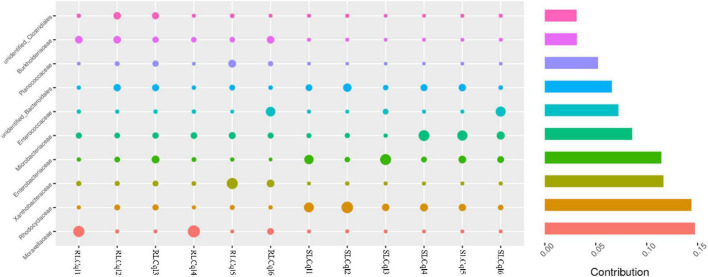
Genus-level contributions in strains of *Culex quinquefasciatus* that are susceptible and resistant to *Lysinibacillus sphaericus* C_3_-41.

Compared with the susceptible strain, the relative abundance of the genera *Rahnella* and unidentified *Rhodocyclaceae* was significantly lower in the resistant strain (Figure 4). In addition, the relative abundance of *Rhodocyclaceae bacterium* MORI9 in the resistant strain was also significantly lower than that in the susceptible strain (Figure 4). The differences in the relative abundances of the symbiotic bacterial communities between the SLCql and RLCql populations suggest that a critical role for gut symbionts in *L*. *sphaericus* C_3_-41 resistance in *Cx. quinquefasciatus*. Furthermore, the *Rhodocyclaceae bacterium* in the six replicates of RLCql demonstrated a lower genus-level contribution compared to SLCql (Figure 5). Taken together, these data suggest that gut symbionts may play an important role in the development of resistance against *L*. *sphaericus* C_3_-41.

## Discussion

Symbiotic gut bacterial communities have been shown to impact several key aspects of host biology, including host nutrition, growth and reproduction, metabolism, immunity, and defense (Engel and Moran, 2013). Therefore, the insect gut microbiome may be considered a virtual organ that plays an integral role in the physiology of the host and is essential for the host’s health (Wang and Qu, 2017). In line with this, this study demonstrated that the symbiotic gut bacterial communities of *Cx. quinquefasciatus* were mainly distributed in the Proteobacteria, Actinobacteria, Firmicutes, and Bacteroidetes phyla (Figures 2A,B). Similarly, previous studies have also identified four main phyla, Proteobacteria, Firmicutes, Bacteroidetes, and Actinobacteria, in *Aedes aegypti* and *Anopheles gambiae* larvae (Coon et al., 2016; Pennington et al., 2016; Guégan et al., 2018). Additionally, Proteobacteria, the most prominent phylum, accounted for approximately 15–79% of the total phyla, indicating that it may play a key role in the gut of *Cx. quinquefasciatus*. Actinobacteria and Bacteroidetes are also likely to be important, as they can impact mosquito development (Coon et al., 2014).

The structure of the symbiotic gut bacterial communities of the resistant strain was different to that of the susceptible strain. The enrichment of different bacterial groups yields a competitive advantage under insecticide selection (Dada et al., 2018). However, to some extent, the composition of the microbial community is affected by the feeding behavior of the larvae (Strand, 2019). During the selection and maintenance of resistance to *L. sphaericus* C_3_-41, the larvae of *Cx*. *quinquefasciatus* would ingest a large amount of *L. sphaericus* C_3_-41, which may alter the microbial diversity and community structure in the gut. In this study, the relative abundances of Firmicutes, Proteobacteria, and unidentified Bacteria in the gut of susceptible *Cx*. *quequinfasciatus* were lower than those in resistant strain (Figures 2A,B), indicating that these phyla may play a critical role in the development of resistance against *L*. *sphaericus* C_3_-41.

Moreover, the observed genus-level variations included significantly attenuated levels of the relative abundance of *Rahnella*, *Rhodocyclaceae bacterium*, and unidentified *Rhodocyclaceae*, and significantly higher levels of the relative abundance of *Xanthobacter*, *Comamonas*, *Leucobacter*, *Pseudochrobactrum*, *Empedobacter*, *Hydrogenoanaerobacterium*, *Sphingobacterium*, unidentified Bacteria, and *Morganella* in the resistant strain. These changes are likely related to the role of the gut microbiome as a functioning organ. To the best of our knowledge, however, these genera have not yet been reported in association with insecticide resistance, especially *Rhodocyclaceae bacterium*. Therefore, further studies are warranted to determine the roles of these bacteria in the mechanisms of insecticide resistance.

## Conclusion

In conclusion, the structural differences in the symbiotic gut bacterial communities between resistant and susceptible *Cx*. *quequinfasciatus* were identified. Insecticide selection was found to significantly alter the composition of gut symbionts in *Cx*. *Quequinfasciatus*, and changes in the bacterial communities may occur after the development of resistance. This study provides a basis for future studies on the mechanisms of resistance to *L*. *sphaericus* C_3_-41 in *Cx*. *quequinfasciatus*, as well as a theoretical basis for in-depth study on the interaction between symbiotic gut bacterial communities and their hosts.

## Data availability statement

The original contributions presented in this study are included in the article/supplementary material, further inquiries can be directed to the corresponding authors.

## Author contributions

XH and ZY conceived and designed the research. XZ and HM conducted the experiments, analyzed the data, and wrote the manuscript. All authors have read, corrected, and approved the manuscript.
